# Safe Use of Adrenaline in Cryosurgery for Eyelid Basal Cell Carcinoma Evaluated With Infrared Thermography

**DOI:** 10.1111/iwj.71005

**Published:** 2026-08-02

**Authors:** Anna H. C. Wiktorin, Bohman Elin, Johanna V. Berggren, John Albinsson, Sheikh Rafi, Malmsjö Malin

**Affiliations:** ^1^ Division of Ophthalmology and Vision, Department of Clinical Neuroscience Karolinska Institutet and St. Erik Eye Hospital Stockholm Sweden; ^2^ Department of Clinical Sciences Lund, Ophthalmology and Skåne University Hospital Lund Lund University Lund Sweden

**Keywords:** adrenaline, basal cell carcinoma, cryosurgery, eyelid, thermography

## Abstract

The efficacy of cryosurgery for skin cancers is believed to depend on thaw time, which may be influenced by the presence of adrenaline in local anaesthetics due to its vasoconstrictive effects. This is the first study to perform detailed, non‐invasive temperature measurements using infrared (IR) thermography during cryosurgery for basal cell carcinomas (BCCs), evaluating the impact of adrenaline on thaw time. A total of 15 patients with eyelid BCCs underwent cryosurgery with local anaesthetics both with and without adrenaline. Thawing was continuously monitored using a high‐resolution IR camera and compared with the surgeon's visual assessment. No significant difference in thaw time was observed between anaesthetics with and without adrenaline. Thermographic mapping revealed a triphasic thawing curve. The surgeon's visual assessment of thawing corresponded well with the point at which tissue temperatures rose above 0°C, likely indicating the onset of reperfusion, thus providing a clinical margin beyond the actual phase transition. This is the first study to use IR thermography to monitor cryosurgery of BCCs, demonstrating that adrenaline in local anaesthetics does not affect thaw time. The findings suggest that deeper tissue perfusion during BCC treatment may counteract superficial vasoconstriction, supporting the effective use of cryosurgery regardless of adrenaline use.

## Key Points

- Infrared thermography enabled real‐time, non‐invasive mapping of tissue temperature during cryosurgery for eyelid basal cell carcinoma.
- The thawing process followed a characteristic triphasic temperature curve, including an extended plateau phase around 0°C.
- Local anesthetics containing adrenaline did not significantly affect thaw time compared with anesthetics without adrenaline.

## 1 Introduction

Surgical excision of basal cell carcinomas (BCCs) of the eyelids can be difficult due to the complex function of the eyelids and neighbouring structures, such as the tear ducts. Cryosurgery, curettage, electrocautery/electrodessication, radiation therapy, photodynamic therapy and immunotherapy [1, 2] are tissue‐sparing alternatives to conventional surgery for cutaneous lesions. Cryosurgery has been used to remove BCCs since the 1960s [3] and is appropriate for well‐defined tumours, especially in the periocular region where the tissue margins to surrounding sensitive structures are narrow [4]. Cryosurgery has been proven to be associated with a low recurrence rate, as well as good cosmetic and functional outcomes [4, 5, 6, 7, 8].

Effective cryonecrosis of malignant tumours typically requires tissue temperatures as low as −50°C [5, 9, 10]. Despite the longstanding clinical use of cryosurgery, detailed imaging and temperature mapping of the procedure remain limited. This is likely due to the historic lack of accurate, non‐invasive temperature monitoring methods. In addition, the role of adrenaline in local anaesthetics, commonly used during cryosurgery, has not been fully elucidated in this context. In a recent study by the authors, infrared (IR) thermography was used to monitor tissue temperature during cryosurgery of eyelid actinic keratosis [11]. The findings revealed significantly longer thaw times when a local anaesthetic with adrenaline was used, suggesting that adrenaline‐induced vasoconstriction may delay reperfusion and prolong the thaw phase [11]. These observations are supported by earlier animal studies, which showed increased necrosis and treatment effect when adrenaline was used prior to cryosurgery [12, 13]. However, unlike actinic keratoses, which are superficial, premalignant lesions confined to the epithelium, BCCs are invasive malignancies that extend into the dermis and potentially deeper tissues, requiring a more extensive and deeper freezing protocol. Whether adrenaline similarly affects thawing dynamics in this context remains unknown. To address this, the present study aimed to perform a detailed mapping of tissue temperature changes during thawing in cryosurgery for eyelid BCCs and to evaluate the potential impact of adrenaline in local anaesthetics on this process.

In clinical practice, the effects and depth dose during cryosurgery are estimated through surrogate parameters such as freeze time, lateral spread, palpation and visual assessment of thaw time [4, 5, 7]. Until recently, there has been no reliable method for accurately monitoring tissue temperature during treatment. Attempts have been made using invasive techniques, such as inserting thermocouple needles into the tissue [5, 7, 8], but these methods only provide point measurements and offer limited information on temperature distribution. Non‐invasive imaging techniques like MRI, ultrasound and CT have been explored but are often impractical due to their complexity and cost. As early as 1996, Pogrel et al. demonstrated that IR thermography could accurately monitor cryosurgical temperatures in animal models, although the technology at the time was too costly and impractical for clinical use [14]. Modern IR cameras, however, have significantly improved in terms of spatial resolution, thermal sensitivity and acquisition speed. They are now compact, affordable and easy to operate, making them suitable for real‐time, non‐invasive temperature mapping in clinical settings. In 2021, Kovalov et al. confirmed the feasibility of using IR thermography to monitor freezing and thawing during cryosurgery in rats [15]. This approach was further validated in a recent study by the authors, where temperature dynamics were successfully mapped during cryosurgery of actinic keratosis in humans. Despite these advancements, no previous studies have applied IR thermography to monitor tissue temperature during cryosurgery of malignant tumours such as BCCs.

The aim of the present study was thus to map the temperature during cryosurgery of BCCs non‐invasively, using a high‐precision IR camera and to evaluate the effect of adrenaline in local anaesthetics. The results were also compared to the surgeon's visual assessment of thaw time. Understanding the influence of adrenaline is crucial, and the outcome will aid oculoplastic surgeons when deciding on the best treatment for BCC lesions in patients wishing to avoid adrenaline or where adrenaline is contraindicated.

## 2 Methods

### 2.1 Ethics

The study was evaluated and approved by the Swedish Ethical Review Authority (2013/825 and 2019‐05603). It was carried out in accordance with the principles laid down in the Declaration of Helsinki as amended in October 2013. All patients gave their fully informed written consent.

### 2.2 Patients

A total of 20 patients with BCCs on their eyelids, referred to the Oculoplastic and Orbital Services at St. Erik Eye Hospital, Solna, Sweden, for cryosurgery, were recruited for the study. Inclusion criteria were well‐defined tumours, confirmed by a 2‐mm punch biopsy without histopathological signs of a highly aggressive growth pattern and located within the orbital rim or in the medial canthal area. Subjects were excluded if they were unable to provide informed consent or if they were not physically or mentally able to cooperate during the procedure under local anaesthesia. Two patients declined participation in the study and one had to be excluded due to a vasovagal reaction during the first freeze–thaw cycle. In total, 15 patients were included in the study, eight male and nine female. The median age was 78 years (range 45–94 years). One participant had skin Type I, nine Type II and five Type III on the Fitzpatrick scale. Five patients had known cardiovascular disease or diabetes Type 2, six of the patients were taking antihypertensive drugs and one anticoagulant medication. Two patients smoked on a daily basis. The tumour location was recorded together with the vertical and horizontal diameters. Five were in the medial canthal area, nine in the pretarsal area and one in the preseptal area of the eyelids. The tumours treated in this study measured between 3 and 17 mm (median 6 mm).

### 2.3 Surgical Procedure

The treatment was carried out by an experienced senior oculoplastic surgeon (co‐author E. B.) during an outpatient visit. The tumour borders were marked. Local anaesthetic drops (Tetrakain 1%, Bausch & Lomb, Stockholm, Sweden) were applied to the surface of the eye and local anaesthetic was infiltrated in the areas (see below). When deemed necessary, a clear plastic Jaeger lid plate (Bausch & Lomb, Saint Louis, MO, USA), thick enough to confine the cooling, was used to protect the eye. During cryosurgery, neoprene cones of various diameters and 3‐mm‐thick walls (Cortex Technology, Aalborg, Denmark) were pressed against the affected tissue. The cone with an inner diameter at the narrowest end that well circumscribes the tumour was used. The cone could be squeezed to fit oval tumours. A liquid nitrogen unit (CryAc, Brymill Cryogenic System, Connecticut, USA or CryoPro, Cortex Technology, Hadsund, Denmark) was used to spray nitrogen openly into the cone. Freezing continued until frost was observed on the skin all around the outer edge of the cone, which took 20–40 s. The cone was left in place until thawing of the tissue spontaneously allowed it to be removed. The mark resulting from pressure from the cone, that is, the treatment zone, could easily be seen on the frozen skin area. The freeze time of the treatment zone was clinically defined as the time until thawing was observed to reach any point on the inner diameter of the treatment zone. A clinical thaw time of at least 1 min was considered desirable. The frozen tissue was allowed to thaw completely, as confirmed by measurements with the IR camera. A second freeze–thaw cycle was then performed. A third freeze–thaw cycle was performed if either of the two thaw times was determined to have been less than 1 min. If cryosurgery involved the eyelid margin, patients were prescribed postoperative application of topical antibiotic ointment in the operated eye for 1 week.

### 2.4 Local Anaesthetic With and Without Adrenaline

Before the first freeze–thaw cycle, a local anaesthetic without adrenaline was injected in the affected area (20 or 10 mg/mL Lidocaine, Mylan AB, Stockholm, Sweden). Thereafter, before the second freeze–thaw cycle, the same area was injected with a local anaesthetic containing adrenaline (Xylocain Dental Adrenalin, 20 mg/mL + 12.5 μg/mL, Dentsply DeTrey GmbH, Konstanz, Germany). The local anaesthetic solutions were preheated to 37°C to avoid effects on perfusion due to cooling of the tissue. The waiting time before surgery was 2 min to achieve the full vasoconstriction effect of the adrenaline [16]. To assess the effect of repeated cryosurgery on thaw time, two control patients, in addition to the 15 patients included in the study, received the same local anaesthetic in both freeze–thaw cycles. One patient was given the anaesthetic with adrenaline (Xylocaine Dental) and the other was given the anaesthetic without adrenaline (20 mg/mL Lidocaine). No noticeable difference in thaw time was observed between the first and second cycles in either case, and this aspect was therefore not further pursued in the study.

### 2.5 IR Camera

A high‐resolution IR camera (FLIRA655sc; FLIR Systems AB, Danderyd, Sweden) was used to monitor cryosurgery. A continuous sequence of digital images was automatically recorded during the entire procedure for observation of the object's thermal field dynamics (thermographic movie). The IR camera was a focal plane array, uncooled microbolometer with 640 × 480‐pixel resolution and thermal sensitivity/NETD (noise equivalent temperature difference) < 30 mK. The IR camera was calibrated in the range −40°C to +150°C. The IR camera was mounted on a camera arm on a movable cart (Flexion‐port 300 mm with extension beam mounted on a Vexio‐cart, ITD GmbH, Munich, Germany) and positioned approximately 50 cm above the tumour. The software ResearchIR 4 (FLIR Systems AB, Danderyd, Sweden) was used for IR image capturing, and postprocessing was performed in MATLAB R2022a.

**FIGURE 1:**
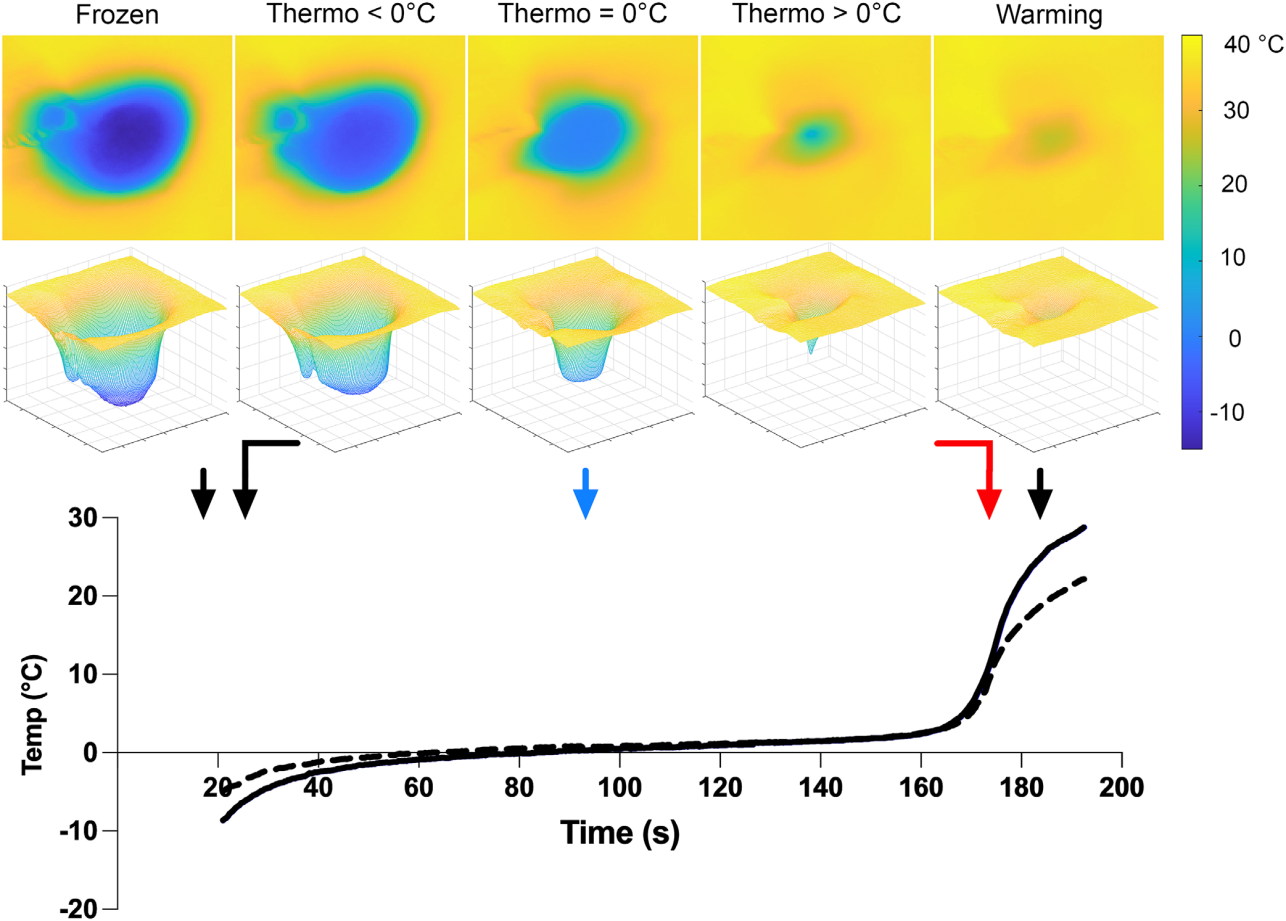
The upper images are representative examples of thermographic mapping of an area of tissue containing a BCC undergoing cryosurgery. They show the tissue when frozen and during the various phases of the thawing period. The time frames correspond to the timescale in the graphs below, which show the triphasic increase in temperature during thawing. The black graph is without, and the dotted graph with adrenaline additive. The arrows indicate the times at which the maps were recorded; the light blue arrow indicates when the tissue temperature reached 0°C (thermo = 0°C), and the red arrow indicates when the temperature increased above 0°C (thermo > 0°C).

The thawing of the treated area was assessed both visually by the surgeon and by thermography. The following times were determined (for schematic illustrations, see Figure 1).

- Time to ‘visual thawing’, that is, the surgeon's visual assessment of thawing based on how the treatment zone and total treated area slowly transformed from a white ice‐ball with dense structure to the same density and colour as the surrounding, untreated tissue.
- The thermographic time point at 0°C is denoted ‘thermo = 0°C’.
- The thermographic time point in the second warming *phase*, that is, when the temperature increased above 0°C, is denoted ‘thermo > 0°C’. This time point was defined as the middle of the second slope of the temperature curve.

### 2.6 Calculations and Statistical Analysis

Thermographic calculations were performed on the recorded pixels of the treated area. Data are presented as median values and ranges. Statistical analysis was performed with the Wilcoxon matched‐pairs test and Kruskal–Wallis test with Dunn's test for multiple comparisons. Significance was defined as *p* ≤ 0.05. GraphPad Prism 10 (GraphPad Software Inc., San Diego, CA, USA) was used for calculations and statistical analysis.

## 3 Results

### 3.1 Thermographic Mapping

The increase in temperature over time measured with thermography followed a triphasic curve. The shape of the curve showed three different stages: (1) an initial rapid increase in temperature from below −40°C (the lowest calibrated value of the IR camera used in this study) to just below 0°C; (2) a plateau phase at a temperature around 0°C, showing a slight linear increase within the period and (3) a subsequent rapid rise in temperature to that of the surrounding tissue. This is in line with the fact that more energy is required in phase transitions, that is, from solid to liquid and from liquid to gas. Figure 1 presents a representative example of the thermographic mapping and the triphasic thawing process.

### 3.2 Visual vs. Thermographic Measurements

**FIGURE 2:**
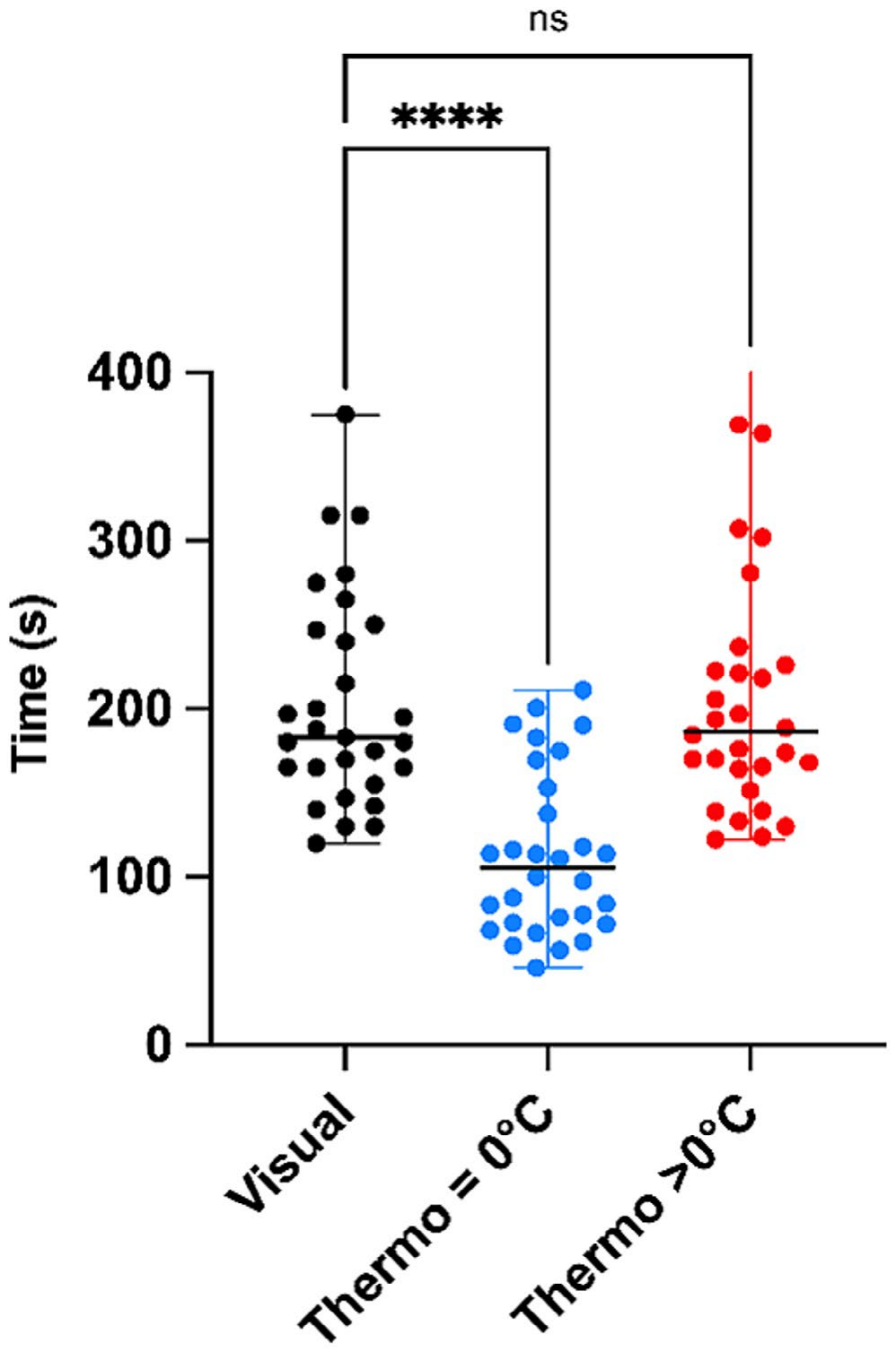
Scatter plots showing the thawing time of tissue undergoing cryosurgery for BCC, as assessed visually by the surgeon (black) and by IR thermography with the thermographic time points thermo = 0°C (blue) and thermo > 0°C (red). The visual assessment was based on the normalisation of density and colour in the treated area. The thermographic time to thawing was measured both when the tissue temperature reached 0°C (thermo = 0°C), and at the start of the second rapid phase of warming, when the temperature increased above 0°C (thermo > 0°C). The graph shows all data from 30 freezing cycles in 15 patients treated with local anaesthetics without and with adrenaline. Note that the surgeon's visual assessment of thawing time agrees well with the second rapid warming phase (thermo > 0°C) measured with IR thermography. A significant difference (*p* < 0.0001) was seen between the surgeon's visual assessment and thermo = 0°C, which implies that temperatures below and around the plateau phase may not be perceptible visually.

The thawing of the tissue and BCC was also assessed visually by the surgeon, based on the normalisation of density and colour in the treated area. The thermographic thawing time was characterised by the time when 0°C was reached (thermo = 0°C) and after the transition phase, the start of rapid warming, when temperatures increased above 0°C (thermo > 0°C). The graph in Figure 2 shows data from 30 freeze–thaw cycles in 15 patients. The data, when using local anaesthetics with and without adrenaline, are combined.

It can be seen that the surgeon's visual assessment of the time at which the tissue had thawed was comparable to the start of the rapid warming phase, that is, when the temperatures of the tissue started to rise above 0°C. However, there was a significant difference (*p* < 0.0001) between the visual assessment and the thermographic time point thermo = 0°C, which shows that temperatures below and around the plateau phase cannot be perceived visually (Figure 2). This significant difference reassures the surgeon that the clinical assessment of thawing is longer than the time taken for the tissue to reach a temperature of 0°C.

### 3.3 Anaesthetics With and Without Adrenaline

**FIGURE 3:**
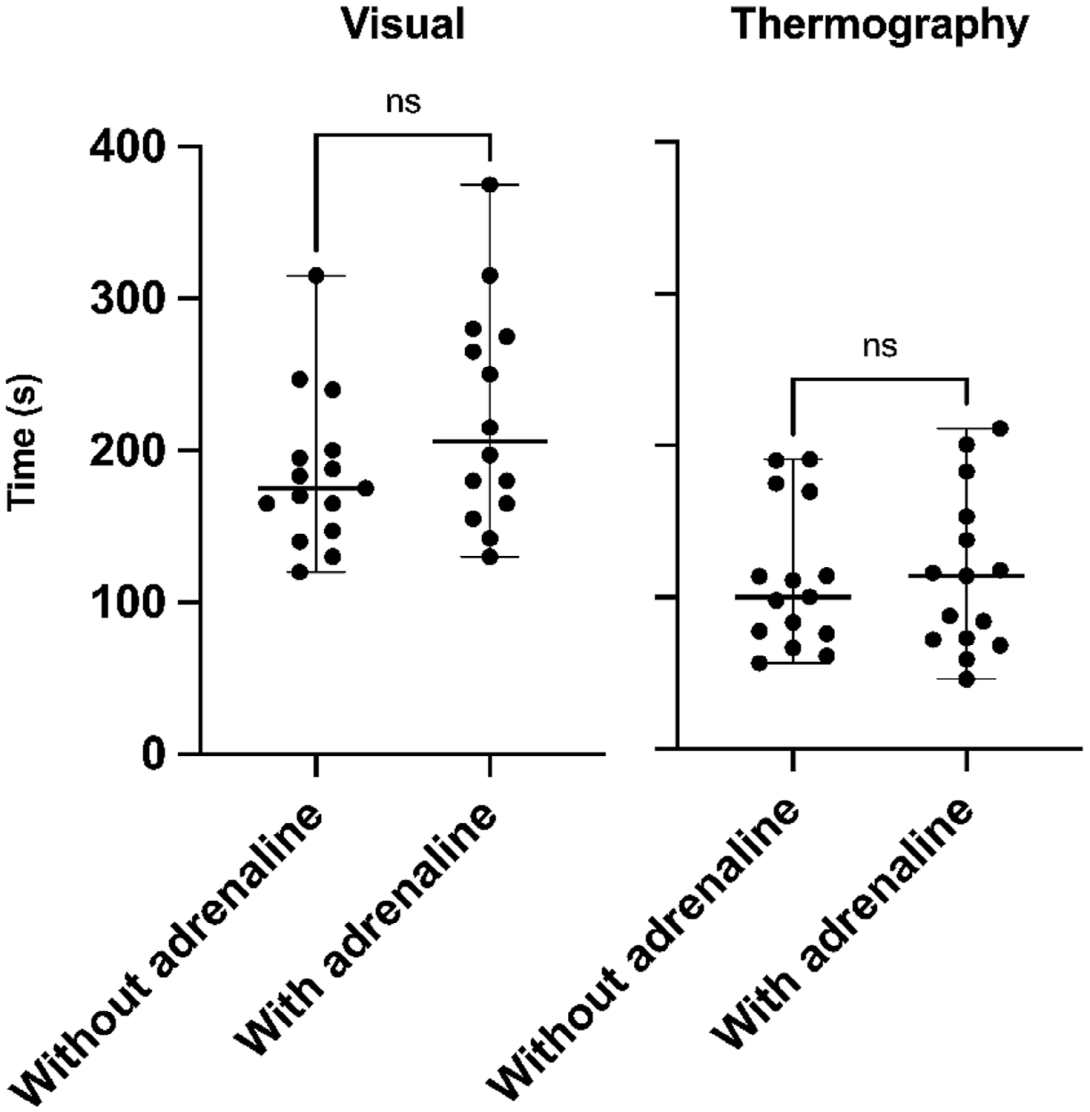
Scatter plots showing the thaw time of tissue undergoing cryosurgery for BCC using local anaesthetic with and without adrenaline. Data from all 15 patients is included. The thaw time was estimated visually by the surgeon and determined by IR thermography. There was no significant difference in the thaw time when using anaesthetic with or without adrenaline, both when estimated visually by the surgeon (*p* = 0.065) and measured by IR thermography (*p* = 0.72).

The thaw time was also measured to compare the two kinds of anaesthetic, that is, with or without adrenaline, both when observed visually and when measured thermographically (Figure 3). There was no statistically significant difference, suggesting a similar effect of the cryosurgical treatment regardless of the type of local anaesthetic used.

## 4 Discussion

This is the first study to monitor tissue temperature during cryosurgery of BCCs using IR thermography while evaluating the impact of adrenaline in local anaesthetics on thawing dynamics. The results show that the presence of adrenaline did not affect thaw time, indicating that the choice of local anaesthetic can be based on the patient's medical condition or subjective preferences rather than concerns about the efficacy of cryosurgery. The present results contradict both our previous study on actinic keratosis, where we showed that thaw time was significantly longer with adrenaline [11] and previous animal studies by Myers et al. and Passy et al., which found that the addition of adrenaline to the local anaesthetic significantly increased the size of the necrotic area after cryosurgery [12, 13]. Furthermore, this challenges the assumption that cryosurgery should always be performed with adrenaline for optimal treatment effect [13].

The discrepancy between the present and the previous study on actinic keratosis cannot be fully explained by the present data but may be attributed to differences in vascular involvement at varying tissue depths. Actinic keratosis is a superficial, premalignant lesion confined to the epithelium; hence, a short and superficial freezing is adequate. In contrast, BCCs are malignant lesions that extend into the entire dermis and sometimes deeper tissues. Consequently, for BCCs, a longer freezing is necessary, involving deeper and more well‐perfused areas. Within the skin, two vascular plexuses exist in the dermis: the upper plexus located in the papillary dermis and a lower network at the dermal‐subcutaneous interface [17]. Furthermore, Bunke et al. demonstrated in their study that an adrenaline injection given at the level of the superficial vascular plexus affected the blood flow in the superficial but not in the deeper vascular plexus [18]. Since the local anaesthetic, in both this and the previous study of actinic keratosis, was injected superficially into the thin periocular skin, it might have resulted in vasoconstriction of the superficial vascular plexus while the deeper vascular plexus remained unaffected. When performing superficial freezing, as for actinic keratosis, only the superficial plexus is involved, leading to a significant difference in thaw time in the presence of adrenaline. However, in the present study of BCCs, a longer freezing time was indicated, thereby involving not only the superficial but also the deeper vascular plexus. Consequently, due to an intact deeper blood flow, more blood perfused the affected area, leading to a shorter thaw time and no difference when using local anaesthetic with or without adrenaline.

The present study supports current clinical practice, where the effect of cryosurgery is commonly estimated by visual assessment of thaw time, that is, the time at which the skin regains its normal or reddish colour after being frozen [4, 5, 7]. The results of the present study showed that the time at which the surgeon determined the tissue had thawed was longer than the time taken for the skin temperature to reach 0°C. This implies that the surgeon's visual assessment determines the time when reperfusion of the treated area starts, rather than when the temperature reaches 0°C. This confirms that the clinical waiting time is considerably longer than that needed for the tissue to reach 0°C, which means that the visual evaluation provides a safe margin for ensuring adequate thawing. Furthermore, IR thermography confirmed the rapid cooling of the treated area after freezing, and the slow triphasic thawing, which is important for the therapeutic effect of cryosurgery. It is believed that the efficiency of cryosurgery depends on the temperature and size of the treated area, the duration of freezing and the rates of the freezing and thawing cycles [10, 19, 20, 21, 22]. Maximal cell necrosis is achieved by a rapid freeze of tissue followed by a slow thaw phase since the combination leads to high intracellular ice crystal formation and extensive electrolyte disturbance [10, 20, 22, 23].

In this study, IR thermography was used to non‐invasively monitor tissue temperature at the surface during cryosurgery. While the technique primarily captures surface temperatures, previous research supports its validity for estimating subsurface freezing effects. In a study by Kovalov et al., IR thermography combined with thermocouple measurements in rats showed that the hemispherical shape and lateral spread of the frozen tissue volume corresponded well with the earlier mathematical assumptions made by Torre [8, 15]. These findings suggest that surface temperature mapping with an IR camera can provide a reliable representation of the overall freezing pattern, supporting the use of thermography as a clinically relevant tool in cryosurgery.

A limitation of the present study is that only temperature was monitored and not clinical outcomes such as induced tumour necrosis. However, since cryosurgery is often selected to avoid surgical incisions, particularly in sensitive areas such as the periorbital region, where structures like the lacrimal drainage system are at risk, it would be ethically and practically challenging to design a study that includes histological assessment of treatment effect in humans. Moreover, such a study would require a large number of patients to achieve sufficient statistical power due to considerable variability in tumour size, anatomical location and vascularisation. However, an additional advantage of a larger cohort would be the ability to more definitively assess the potential impact of adrenaline in local anaesthetics. Although the present study showed no significant difference in thaw time between anaesthetics with and without adrenaline, a larger sample size would strengthen the power and help ensure that a subtle effect is not being overlooked. On the other hand, since there was no observable trend in thaw time differences between adrenaline and no adrenaline, this difference is likely so small that achieving statistical significance would require a considerable sample size, which would not be clinically attainable. Furthermore, the difference is likely so minor that it does not exclude cryosurgery as a treatment option for patients who wish to avoid adrenaline or for those who, due to medical reasons, should avoid adrenaline.

In addition, larger tumours require longer freeze times, which could potentially influence both thaw duration and the effect of adrenaline. However, due to the limited sample size in the present study, the impact of tumour size on thaw time could not be evaluated. The BCCs included ranged from 3 to 17 mm in diameter, and although larger lesions are expected to thaw more slowly and may be more affected by adrenaline, this could not be systematically assessed. For this reason, comparisons of adrenaline effects were not made between individuals. Instead, paired comparisons were performed within each patient between the first and second freeze–thaw cycles. A larger clinical study would be necessary to investigate the influence of tumour size on the effect of adrenaline in local anaesthetics.

## 5 Conclusions

In conclusion, this study is the first to use IR thermography to map tissue temperature during cryosurgery of BCCs and evaluate adrenaline's effect in local anaesthetics. The presence of adrenaline in local anaesthetics did not have any effect on the thawing time. This is likely due to deeper freezing engaging both superficial and deep vascular plexuses, with deeper tissue perfusion compensating for superficial vasoconstriction. These findings support the effective use of cryosurgery regardless of whether adrenaline is contraindicated.

## Funding

This study was supported by the Karolinska Institute Foundation for Eye Research, the Swedish Cancer Foundation, Elsy, Harry and Henrik Johansson's foundation, Mrs. Berta Kamprad's Foundation, the Sjöberg Foundation, the Lund University grant for research infrastructure, the Swedish Government Grant for Clinical Research (ALF), Skåne University Hospital (SUS) research grants, Skåne County Council research grants, Crown Princess Margaret's Foundation (KMA), Friends of the Visually Impaired Association in the County of Gävleborg, the Foundation for the Visually Impaired in the County of Malmöhus, Carmen and Bertil Regnér's Foundation, IngaBritt and Arne Lundberg's Research Foundation, the Swedish Eye Foundation, the Cronqvist Foundation, the Swedish Medical Association and the Edvin Giströms Foundation.

## Ethics Statement

The study was approved and an ethical permit granted by the Swedish Ethical Review Authority (2013/825 and 2019‐05603). The study was conducted in accordance with the ethical guidelines of the Declaration of Helsinki and all subjects gave their written informed consent prior to inclusion in the study.

## Conflicts of Interest

The authors declare no conflicts of interest.

## Data Availability

The data that support the findings of this study are available on request from the corresponding author. The data are not publicly available due to privacy or ethical restrictions.
